# The role of attitudes towards contradiction in psychological resilience: the cortical mechanism of conflicting resolution networks

**DOI:** 10.1038/s41598-024-51722-3

**Published:** 2024-01-18

**Authors:** Zai-Fu Yao, Meng-Heng Yang, Cheng-Ta Yang, Yun-Hsuan Chang, Shulan Hsieh

**Affiliations:** 1 College of Education, National Tsing Hua University, Hsinchu City, 30013 Taiwan; 2 Research Center for Education and Mind Sciences, National Tsing Hua University, Hsinchu City, 30013 Taiwan; 3 Basic Psychology Group, Department of Educational Psychology and Counseling, National Tsing Hua University, Hsinchu City, 30013 Taiwan; 4 Department of Kinesiology, National Tsing Hua University, Hsinchu City, 30013 Taiwan; 5 Cognitive Electrophysiology Laboratory: Control, Aging, Sleep, and Emotion (CASE), Department of Psychology, National Cheng Kung University, Tainan City, 70101 Taiwan; 6 Graduate Institute of Mind, Brain, and Consciousness, Taipei Medical University, Taipei, Taiwan; 7 Department of Psychology, National Cheng Kung University, 1 University Road, Tainan City, 70101 Taiwan; 8 Institute of Gerontology, National Cheng Kung University, Tainan City, 70101 Taiwan; 9 Institute of Genomics and Bioinformatics, National Chung Hsing University, Taichung, 402 Taiwan; 10 Institute of Allied Health Sciences, National Cheng Kung University, Tainan City, 70101 Taiwan; 11 Department of Public Health, National Cheng Kung University, Tainan City, 70101 Taiwan

**Keywords:** Brain, Cognitive neuroscience, Social behaviour, Social neuroscience, Stress and resilience, Human behaviour

## Abstract

Managing contradictions and building resilience help us overcome life's challenges. Here, we explored the link between attitudes towards contradictions and psychological resilience, examining the role of cortical conflict resolution networks. We enlisted 173 healthy young adults and used questionnaires to evaluate their cognitive thinking styles and resilience. They underwent structural and functional magnetic resonance imaging scans. Our results revealed that contrasting attitudes toward contradictions, formal logic, and naïve dialecticism thinking styles corresponded with varying degrees of resilience. We noted structural and functional differences in brain networks related to conflict resolution, including the inferior frontal and parietal cortices. The volumetric variations within cortical networks indicated right-hemispheric lateralization in different thinking styles. These findings highlight the potential links between conflict resolution and resilience in the frontoparietal network. We underscore the importance of frontoparietal brain networks for executive control in resolving conflicting information and regulating the impact of contradictions on psychological resilience.

## Introduction

Adversity is an inherent aspect of life, impacting people to varying degrees. While some individuals can quickly recover from setbacks, others find it challenging. This perspective is closely tied to the concept of psychological resilience, which empowers individuals to navigate life's ups and downs with balance and composure. Attitudes towards contradictions, as proposed by Peng and Nisbett^1^, offer valuable insights into an individual's mindset and, consequently, their capacity for psychological resilience^1^. Contradictions are an intrinsic part of life, presenting challenges that can be viewed as either insurmountable obstacles or opportunities for personal growth. People with a holistic thinking style tend to be more open to embracing contradictions and often possess a more flexible and adaptive mindset compared to those with a more analytical thinking style, which may involve elements of naïve dialecticism and formal logic^2,3^. This predisposition can be evaluated using the Analysis-Holism scale (AHS)^1,3^. The subscales employed in the AHS for evaluating attitudes toward contradictions unveiled noticeable distinctions between the two contemplated thinking styles (i.e., formal logic vs. naïve dialecticism).

Furthermore, alongside the AHS, recent cross-cultural studies have revealed a noteworthy connection between an individual's capacity for psychological resilience and traditional Chinese thinking styles, such as the Zhongyong thinking scales (ZTS)^4,5^. These studies, as outlined in references 4 and 5, underline the significance of balance, harmony, and flexibility in effectively handling conflicting situations. In examining the role of resilience among East Asian populations, one must consider assessments that encapsulate varying cognitive thinking perspectives, specifically the dialectical and logical approaches as interpreted by the AHS, and the continuum of holistic to analytical thought processes as indicated by ZTS^4,5^. For example, in the Eastern Asian context^6^, a dialectical perspective may be more conducive to resilience, which embraces multiple viewpoints and their integration, may foster resilience. Furthermore, a recent study^7^ that explored the structural relationships between Zhongyong, dispositional mindfulness, resilience, and subjective well-being. This study employed structural equation modeling on a sample of 1099 Chinese high school students and provided robust empirical evidence supporting a positive relationship between Zhongyong and resilience. Specifically, the study found that Zhongyong positively predicts resilience, life satisfaction, and positive affect, while also being a significant mediator in the relationship between dispositional mindfulness and these outcomes. These findings not only align with our theoretical assertions but also reinforce the empirical link between Zhongyong thinking style and psychological resilience. Given the hypothesized alignment between ZTS and naïve dialecticism in their comprehensive and integrative outlooks, it becomes imperative to delve into their interrelations.

One potential link between ZTS and AHS in psychological resilience from Chinese culture prospect is the ability to resolve conflicts and adapt to stress, as effective conflict resolution and adaptability are key factors in developing resilience and managing life's adversities. Yang et al. (2016) have reported that Zhongyong thinking is associated with lower mental distress indicators such as anxiety and depressive symptoms^8^, and higher subjective well-being indicators like self-esteem and life satisfaction, indicating that Zhongyong thinking could be integrated into emotion regulation strategies, with potential applications in therapy to encourage individuals to consider multiple perspectives, think holistically, acknowledge emotional complexity, and maintain interpersonal harmony. Conflict resolution is also one of the factors for promoting resilience in situations of life's difficulties as it can be a source of stress and negative emotions^9–11^. When individuals are in a conflict situation, their brains are faced with competing demands or goals, and cognitive control allows them to prioritize and focus on the most important or relevant goal while inhibiting or suppressing other, less relevant goals or responses. Conflict resolution can be thought of as a specific application of cognitive control in that it involves using cognitive control processes to regulate thoughts and behaviors to resolve a conflict^12,13^. Previous neuroimaging studies have investigated the neural correlates of conflict resolution and the attitude toward contradictions in cognitive thinking styles. For instance, the dorsal attention network is involved in allocating attention and selecting relevant information^14–16^. It consists of a network of brain regions, including the prefrontal cortex, parietal cortex, and superior colliculus, which play critical roles in conflict resolution.

### Overview of the present research

In the current study, we aim to investigate the relationship between the attitude toward contradictions, conflict resolution-related cortical brain networks, and psychological resilience in a sample of young healthy individuals. Specifically, we examined the neural correlates of psychological resilience in different attitudes toward contradictions, and conflict resolution-related cortical brain networks using functional magnetic resonance imaging (fMRI). Neuroanatomical and functional variations in the brain have been linked to diverse cognitive and behavioral tendencies. For instance, variations in certain brain regions have been associated with cognitive and emotional processing differences, which could influence individuals’ resilience capacities^17,18^. We further hypothesized that people with a dialectical thinking style would be associated with differences in brain regions linked to conflict resolution, such as the inferior frontal regions and parietal areas. These cortical mechanisms associated with conflict resolution play a crucial role in psychological resilience. To test these hypotheses, we recruited a sample of young healthy individuals and administered self-report measures to assess their attitude toward contradictions and psychological resilience. We adopted Resilience Scale for Adults (RSA)^19^ to reflects individuals’ psychological resilience, we also used advanced fMRI analysis techniques to examine the relationship between the attitude toward contradictions (as measured by AHS), conflict resolution associated cortical brain networks, and psychological resilience at the neural level. Both brain metrics and resilience scores can be thought of as emergent indexes influenced by a plethora of genetic, environmental, and experiential factors^20^. Moreover, by incorporating the Montreal Cognitive Assessment (MoCA)^21^, the Quality of Life scale (QOL)^22^, and Beck's Depression Inventory-II (BDI-II)^23^ as confounding variables in the investigation of psychological resilience is imperative as these tools collectively measure cognitive function, depressive symptoms, and overall life satisfaction, which can significantly influence individual differences in attitudes toward contradiction and thus potentially modulate the expression of psychological resilience. Our hypothesis extends to anticipate that the correlations observed in holistic and analytic cognitive processes within the framework of ZTS will manifest distinctively when compared to those of naïve dialecticism and formal logic. This is especially pertinent in an East Asian context, where cultural nuances profoundly influence cognitive styles. We expect that an Asian sample will reveal nuanced interplays between these thinking styles, offering insights into how cultural contexts can shape cognitive processing. The anticipated differences in correlations among these thinking paradigms will strengthen our understanding of the cognitive diversity shaped by cultural and philosophical doctrines. Thus, while our model proposes a directional influence from brain metrics to attitudes and subsequently to resilience, this conceptualization is a working hypothesis, open to refinement based on accumulating evidence.

## Methods and results

### Methods

#### Participants

A total of 173 right-handed individuals without any prior psychological or neurological disorders were recruited through online recruitment methods and advertisements on bulletin boards on the campus. All participants aged between 20 and 30 years old with a mean age of 22.57 ± 2.43 years (standard deviation, SD). All participants were given a written informed consent form approved by the Research Ethics Committee (No. 109–419) and Institute of Review Board (IRB, JA-109–95) of the Hospital and signed to agree to participate in this study. Prior to scanning room, all participants filled out behavioral questionnaires concerning thinking styles: AHS^2^ and ZTS^24^; Resilience Scale for Adults (RSA)^19^; mental health status: cognitive status (MoCA)^21^, quality of life (QOL)^22^ and depressive status (BDI-II)^23^ before the brain imaging scanning session. After MRI scans and questionnaires were completed, all participants received USD $80. All procedures performed in studies involving human participants were in accordance with the ethical standards of the institutional and/or national research committee and with the 1964 Helsinki declaration and its later amendments or comparable ethical standards.

#### Measurements of mental health status

The Montreal Cognitive Assessment (MoCA)^21^ is a widely used screening tool to assess cognitive impairment. It was designed to detect mild cognitive impairment (MCI) and early dementia. MoCA consists of a brief 10-min cognitive screening test that is used by healthcare professionals, such as doctors, to quickly assess cognitive impairment in patients. The test consists of a series of tasks that assess various cognitive functions such as drawing, memorization, attention, language, and abstraction. It has been shown to be more sensitive than the Mini-Mental State Examination (MMSE)^25^ in detecting mild cognitive impairment and early dementia. The MoCA is generally considered especially important due to its sensitivity and specificity in detecting MCI, a condition that may precede or accompany mental health disorders, including dementia and depression^26^.

Beck's Depression Inventory-II (BDI-II)^23^ is a self-report questionnaire designed to assess the severity of depression in adults and adolescents aged 13 years and older. It consists of 21 items that measure symptoms of depression, such as sadness, loss of interest in activities, changes in appetite and sleep patterns, feelings of guilt or worthlessness, and thoughts of self-harm or suicide. Scores on the BDI-II range from 0 to 63, with higher scores indicating more severe symptoms of depression. The BDI-II is justified as a key instrument in mental health due to its strong psychometric properties, which include its high reliability, validity, and sensitivity to changes in depression levels over time^27^.

The Quality of Life scale (QOL)^22^ is designed to capture subjective evaluations of an individual's life satisfaction and assess their sense of fulfillment and happiness, including physical health, psychological well-being, social relationships, and environmental factors. Participants were asked to rate their level of satisfaction on a scale from 1 to 7, where 1 indicates very dissatisfied and 7 indicates very satisfied. The scale typically consists of 16 to 17 items that cover the different domains of life satisfaction. The inclusion of Quality of Life (QOL) assessments in mental health evaluations involves the subjective evaluation of both positive and negative aspects of life^28^.

#### A measure of Thinking styles (attitudes towards contradiction)

The AHS was used to assess an individual’s analytic and holistic thinking style^2^ (traditional Chinese version validated by)^29^. The AHS is a self-reported questionnaire with 24 items and four subscales (causality, attitude toward contradictions, perception of change, and locus of attention). Each item is scored on a seven-point Likert-type scale [from 1 (strongly disagree) to 7 (strongly agree)]. In this study, we are most interested in the “attitude toward contradictions” of the subscales calculated from four items’ points. Higher scores indicate a naïve dialecticism thinking style, and lower scores indicate a more formal logical thinking style. East Asian is often described as exhibiting naïve dialecticism as a thinking strategy to tolerate contradictions, displaying lesser inclination to resolve inconsistencies^1^.

#### A measure of thinking styles (Zhongyong belief-value scales)

The ZTS^24^ is based on a set of belief-value scales that help individuals achieve balance, harmony, and moral integrity in their thoughts and actions. These scales include the scale of balance, the scale of harmony, the scale of integrity, and the scale of self-cultivation. Higher scores on the Zhongyong belief-value scales generally indicate that an individual is more likely to exhibit a thinking style that is balanced, harmonious, and grounded in moral integrity and self-cultivation.

#### A measure of psychological resilience

The Resilience Scale for Adults (RSA)^19^ is a self-report questionnaire that was then revised in 2006 ^30^. We adopted a translated Chinese version of RSA; this version of RSA, consisting of 29 items, was scored using a seven-point semantic differential scale, with a higher score indicating greater resilience. The Chinese version has received great reliability of 0.89 using exploratory factor analysis (EFA), and five subscales were as follows: (1) personal strength (RSA_ps), (2) family cohesion (RSA_fc), (3) social resources (RSA_sr), (4) social competence (RSA_sc), and (5) future structured style (RSA_fss).

#### Magnetic resonance imaging (MRI) acquisition parameters

MRI images were acquired by a General Electronic (GE) MR750 3 T scanner (GE Healthcare, Waukesha, WI, USA) at the University. High-resolution structural images with high resolution were obtained with a fast-spoiled gradient-recalled echo sequence including 166 axial slices (repetition time (TR) = 7.6 ms; echo time (TE) = 3.3 ms; flip angle = 12°; field of view (FOV) = 22.4 × 22.4 cm2; matrices = 224 × 224; slice thickness = 1 mm). The entire process lasted for 3 min 38 s. Resting-state functional images were acquired with an interleaved gradient-echo planar imaging pulse sequence (TR = 2000 ms, TE = 30 ms, flip angle = 77, 64 × 64 matrices, FOV = 22 × 22 cm2, slice thickness = 4 mm, no gap, voxel size = 3.4375 mm × 3.4375 mm × 4 mm, 32 axial slices covering the entire brain). There were 245 volumes acquired (The protocol will discard the first five dummy scans to bring the magnetization system to a steady state). During a resting state functional scan, we instructed participants to remain awake, eyes open, and fixated on a white cross displayed on the screen, and the entire scanning time lasted for 8 min and 10 s per participant.

#### General procedures

##### Resting-state functional MRI (rfMRI)

The CONN toolbox 18a (www.nitrc.org/projects/conn) and SPM 12 (http://www.fil.ion.ucl.ac.uk/spm) of Matlab (The MathWorks, Inc., Natick, MA, USA) were used for the preprocessing function images. For detailed parameters and procedures, please refer to earlier studies from our group^31^. To identify functional networks and their properties, we employed a whole-brain parcellation template to define cortical regions of interest (ROI)^32^ and to compute functional connectivity between these ROIs. This template contains 400 brain area nodes, which can be further divided into Yeo’s 17 networks^33^ (see https://surfer.nmr.mgh.harvard.edu/fswiki/CorticalParcellation_Yeo2011 for more detail). Network indexes were calculated for each node, including within-module-degree (WMD) and participation coefficient (PC)^34^. Specifically, PC can be estimated as the degree to which a node is connected to external networks, with values ranging from 0 to 1. Nodes associated solely with other nodes within a single network would have a PC of 0, while nodes with many distributed associates with many different networks would have a PC closer to 1. WMD scores of each network’s ROI were calculated using the mean and SD of the within-network degree (number of intra-network connections) calculated from each functional cortical network. Voxels within a network that have higher WMD values indicate a greater number of connections within the network. By averaging the nodes within each defined network, we extracted PC and WMD values for each network, respectively.

#### T1-weighted structural MRI (sMRI)

We used FreeSurfer 5.31 with an automated surface-reconstruction scheme to estimate GMVs. Regions of interest (ROIs) were extracted using neuroanatomical labels in the Desikan–Killiany Atlas to map on a cortical surface model. GMVs in each ROI of FreeSurfer’s Atlas were extracted from output aparc.stats files.

### Statistical analysis

We hypothesized that cognitive thinking style measurements could be linked to levels of psychological resilience in attitudes toward contradictions. In order to test this, we calculated the AHS_contradiction score and grouped participants into two categories based on Median Split^35,36^ of AHS_contradiction score (for detailed distribution of AHS scores on the formal logic and naïve dialecticism, see the Supplementary file: Fig. 1–2): those with a formal logic thinking style and those with a naïve dialectism thinking style. We then employed a t-test to examine the group differences in demographic information, structural and functional brain metrics between the two groups, including RSA, MoCA, BDI, QOL, and ZTS scores. In addition to the p-value, we also report the Bayes Factor, which may be interpreted as proportional evidence for the presence or absence of an effect. When exploring the functional brain metrics (brain network) differences between the two groups, we used multivariate analysis of variance (MANOVA) to consider all nodes within the network metrics simultaneously. Next, we analyzed the correlation between RSA and questionnaire scores, as well as the correlation with brain metrics that showed significant differences between groups while controlling for gender to minimize potential confounds. To understand the relationship between the brain, attitudes toward contradiction, and resilience, we conducted a mediation analysis to examine the indirect effect. Maximum likelihood estimation and bootstrapping methods were used to estimate the model. The significance of indirect effects was assessed with a 95% confidence interval. Bias-corrected percentile bootstrap estimation, which was used to estimate confidence intervals. 5000 bootstrap iterations were performed. A two-sided *p* < 0.05 was used to reject the null hypothesis if the interval did not include zero. To minimize potential confounds, statistical analysis was first to ensure all participants’ mental health status of demographic information (e.g., MoCA and BDI-II) was identical between groups of contradictory attitudes and even controlled for possible gender effects throughout the entire statistical testing. These strategies help increase the power of this study, which is essential for detecting true effects and reducing the likelihood of false positives. All analyses were conducted using statistical modeling programs SPSS version 22.0., Chicago, IL, USA, network visualization package version 1.4.99.9004 (https://r.igraph.org/) with R^37^ and JASP version 0.15^38^. Mediation analysis was conducted using the PROCESS macro v3.1 developed by Hayes and colleagues^39,40^.

### Transparency and openness

In accordance with Journal Article Reporting Standards (JARS)^41^, we provide details on how we arrived at our sample size, as well as any data exclusions, manipulations, and measures used in our study. All analyses were conducted using statistical modeling programs SPSS version 22.0., Chicago, IL, USA, and JASP version 0.15^38^ and network visualization package version 1.4.99.9004 (https://r.igraph.org/) with R^37^. Moreover, we did not pre-register the analysis or design of this study. An a priori power analysis was conducted using G*Power version 3.1.9.4^42^ to determine the minimum sample size required to test the study hypothesis. Results indicated the required sample size to achieve 80% power for detecting a medium effect, at a significance criterion of α = 0.05 (two-tailed), was a total sample size of N = 128 for the t-test and a total sample size of N = 94 for MANOVA.

### Ethics approval

All participants were given a written informed consent and signed to agree to participate in this study. This study was approved by the Research Ethics Committee (No. 109–419) and Institute of Review Board (IRB, JA-109-95) of Hospital.

## Results

### Demographic information

Participants with formal logic (mean scores = 25.41 ± 4.01) were compared to those with naïve dialecticism (mean score = 34.43 ± 2.41) based on attitude towards contradiction scores showed significant group differences on RSA_fc (*t*(171) = 4.334, *p* < 0.001), RSA_total(*t*(171) = 2.687, *p* < 0.01), QOL_soc (*t*(171) = − 2.569,* p* < 0.05), and ZTS (*t*(171) = − 4.703, *p* < 0.001). On the other hand, demographic measures (i.e., MoCA, BDI-II, QOL_Phy, QOL_Psy, QOL_Env) showed no statistical differences between formal logic and naive dialecticism groups. All results are reported in Table 1.

**Table 1:** Demographic information across participants.

	Formal Logic (N = 82; 43 females, 39 males)		Naïve Dialecticism (N = 91; 52 females, 39 males)		T-test	
	Mean	SD	Mean	SD	*p*-value	BF10
Age	22.707	2.472	22.440	2.400	0.471	0.210
RSA_ps	27.061	6.115	28.275	6.541	0.211	0.342
RSA_fc	32.171	7.328	36.681	6.358	0.000	731.767
RSA_sr	41.878	7.884	44.242	7.709	0.048	1.026
RSA_sc	18.878	5.017	19.275	5.352	0.617	0.185
RSA_fss	18.207	5.166	18.956	5.653	0.366	0.241
RSA_total	138.195	21.519	147.429	23.465	0.008	4.499
MoCA	28.293	1.681	27.978	1.686	0.221	0.331
BDI-II	9.573	8.494	8.143	7.930	0.254	0.303
QOL_Phy	14.070	2.463	14.637	2.119	0.105	0.563
QOL_Psy	12.732	3.978	13.055	2.657	0.527	0.198
QOL_Soc	13.329	2.445	14.231	2.171	0.011	3.396
QOL_Env	14.570	2.025	15.053	1.820	0.100	0.582
ZTS	5.302	0.744	5.811	0.680	0.000	3026.534

RSA: Resilience Scale for Adults (ps: personal strength, fc: family cohesion, sr: social resource, sc: social competence, fss: future structured style), MoCA: Montreal Cognitive Assessment, BDI-II: Beck’s Depression Inventory-II, QOL: Quality of life (Phy: physical health, Men: mental health, Soc: social relations scale, Env: environmental health scale, ZTS: Zhongyong Thinking Scale).

### Group differences between brain metrics: t-test and MANOVA

Between groups from different attitudes were compared, their structural brain metrics in both hemispheres showed significant differences in rostral parts of the inferior frontal gyrus (i.e., Pars Orbitalis in the left (*t*(171) = 3.920, *p* < 0.001, BF_10_ = 166.545) and in the right hemisphere (*t*(171) = 2.317, *p* < 0.05, BF_10_ = 1.942)), Inferior Parietal in the right hemisphere (*t*(171) = 2.162, *p* < 0.05, BF_10_ = 1.416), Middle Temporal in the right hemisphere *(t*(171) = 2.065, *p* < 0.05, BF_10_ = 1.175), Pars Opercularis in the right hemisphere (*t*(171) = 2.104, *p* < 0.05, BF_10_ = 1.264), Precentral in the right hemisphere *(t*(171) = 2.725, *p* < 0.01, BF_10_ = 4.925) and Precuneus in the right hemisphere *(t*(171) = 2.461, *p* < 0.05, BF_10_ = 2.656). Results are reported in Supplementary Table 1.

**Figure 1:**
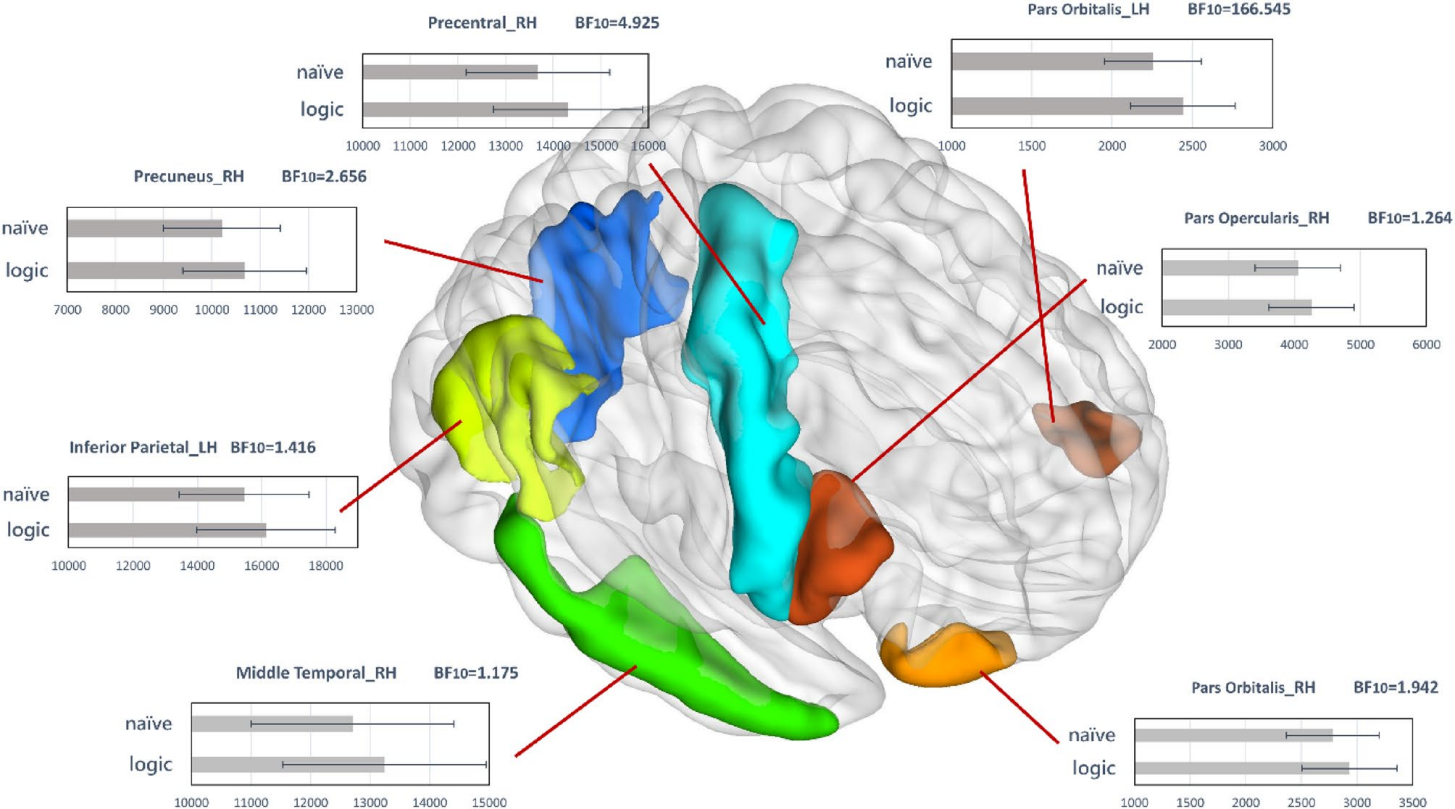
Group differences of brain metrics in the different attitudes towards contradictions. RH: right hemisphere, LH: left hemisphere.

MANOVA analysis was used to examine between-group comparison on functional brain metrics in both hemispheres, showing significant differences in WMD of the central visual network (*F*(24, 148) = 1.710, Pillais’ Trace = 0.217, *p* < 0.05), PC of dorsal attention B (*F*(25, 147) = 1.710, Pillais’ Trace = 0.224, *p* < 0.05), WMD of salience/ventral attention A (*F*(34, 138) = 2.029, Pillais’ Trace = 0.333, *p* < 0.01), and WMD of control C network (*F*(12, 160) = 1.927, Pillais’ Trace = 0.129, *p* < 0.05). Results are reported in Supplementary Table 2. Significant between-group differences in brain metrics are summarized in Fig. 1.

### Correlation between resilience measures and demographic information by group: pearson’s coefficients

**Figure 2:**
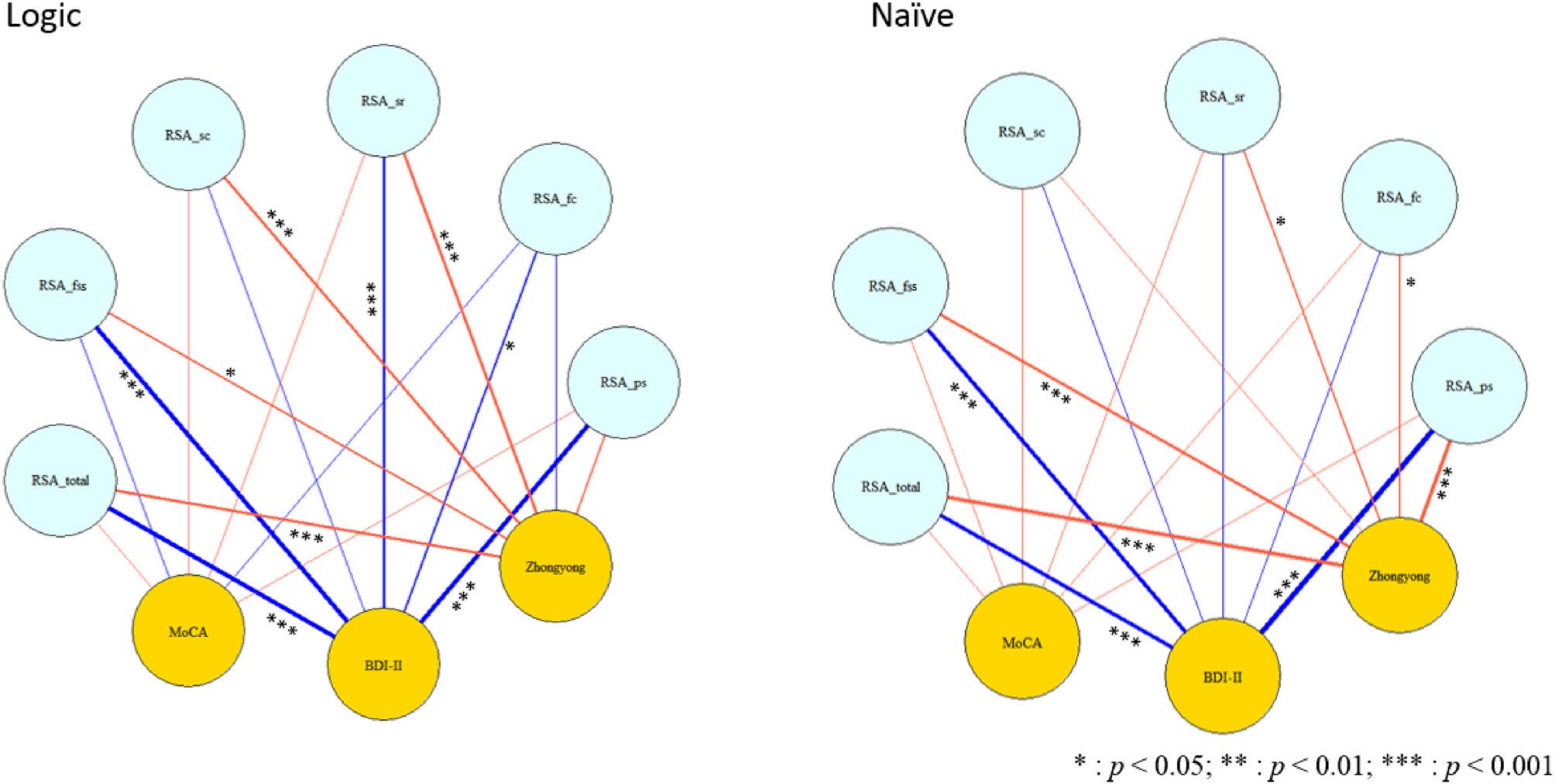
Correlation (Pearson’s r value) between resilience measures and demographic variables by group. RSA: Resilience Scale for Adults (ps: personal strength; fc: family cohesion, sr: social resource, sc: social competence, fss: future structured style), MoCA: Montreal Cognitive Assessment, BDI-II: Beck’s Depression Inventory-II. Blue lines represent a negative correlation, while red lines represent a positive correlation, Zhongyong: Zhongyong thinking styles.

Correlation analysis was performed to explore the relationship underlying behavioral measures. Results are plotted in Fig. 2 and reported in Supplementary Table 3. A significant negative correlation between total resilience scores with BDI-II was observed in formal logic (Pearson’s *r* = − 0.547; *p* < 0.001) and naïve dialecticism (Pearson’s *r* = − 0.419;* p* < 0.001). Moreover, a significant positive correlation between AHS_contradiction and Zhongyong scores (Pearson’s* r* = 0.360;* p* < 0.001) across groups was observed. Similarly, we also observed a significant positive correlation between RSA_sr and QOL_soc in both groups (formal logic: Pearson’s *r* = 0.634;* p* < 0.001; naïve dialecticism: Pearson’s *r* = 0.510;* p* < 0.001).

### Correlations between resilience measures and brain metrics by group: Pearson’s coefficients

**Figure 3:**
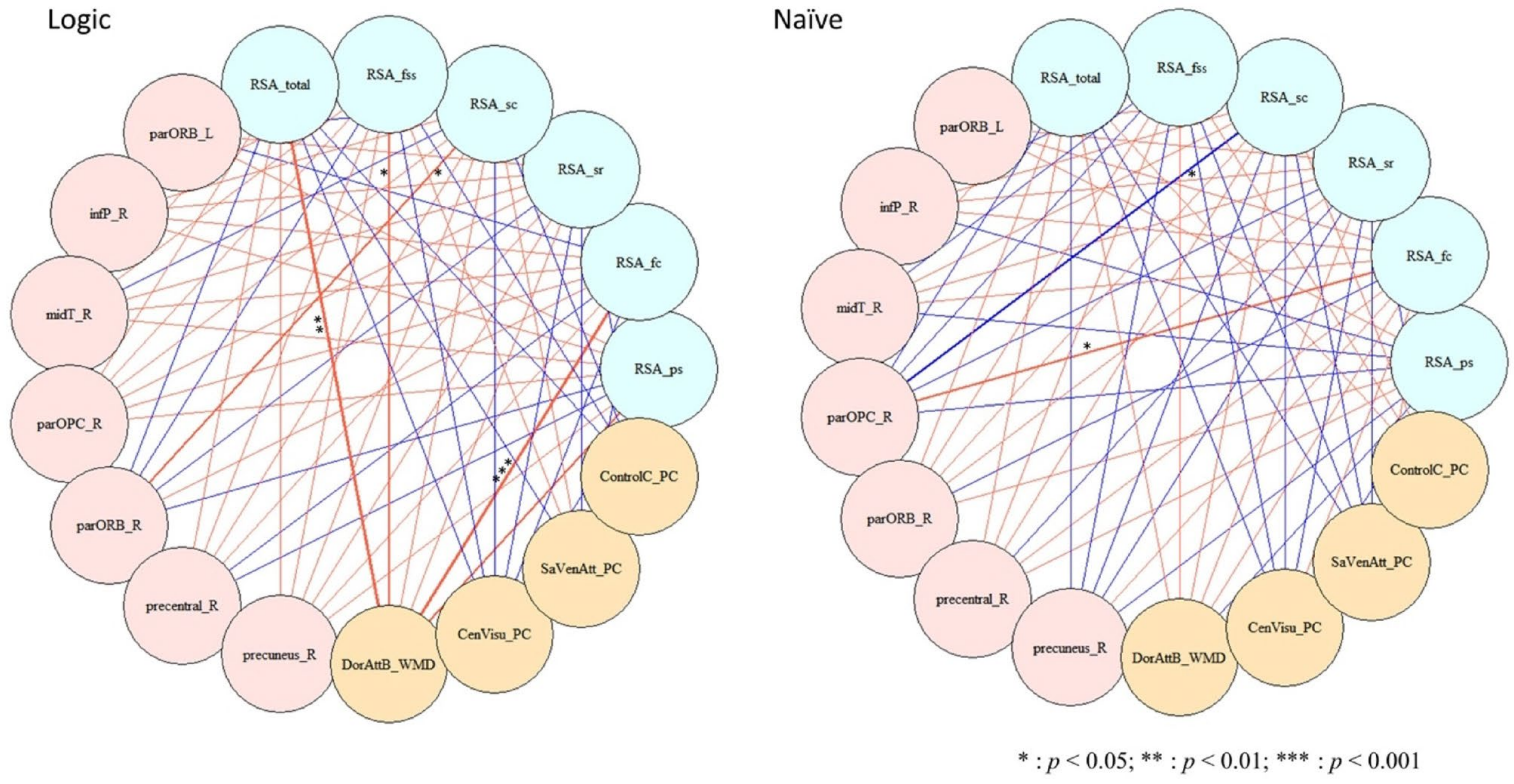
Correlations between resilience measures and brain metrics by group. RSA: Resilience Scale for Adults (ps: personal strength; fc:family cohesion; sr: social resource; sc: social competence; fss: future structured style); parORB: Pars Orbitalis; infP: Inferior Parietal; midT: Middle Temporal; parOPC: Pars Opercularis; DorAtt: dorsal attention; CenVisu: central visual; SaVenAtt: Salience/Ventral Attention; WMD: Within module degree; PC: participation coefficient; Blue lines represent negative correlation, while red lines represent positive correlation.

Correlation analysis explored the relationship between behavioral measures and brain metrics. For the formal logic group, significant positive correlations between brain metrics of dorsal attention networks and subscales of resilience score were observed. Specifically, positive correlations of DorAttB_WMD with RSA_fc (Pearson’s *r* = 0.222; *p* < 0.01), RSA_sr (Pearson’s *r* = 0.161; *p* < 0.05), RSA_sc (Pearson’s *r* = 0.155; *p* < 0.05), and RSA_total (Pearson’s *r* = 0.214;* p* < 0.01) were observed, whereas a negative correlation between RSA_sc and parORB_L (Pearson’s* r* = − 0.250; *p* < 0.05) was also observed. For the naïve dialecticism group, a positive correlation between parOPC_R and RSA_fc was found (Pearson’s *r* = 0.209; *p* < 0.05), while a negative correlation between parOPC_R and RSA_sc was observed (Pearson’s *r* = − 0.223; *p* < 0.05). Results were plotted in Fig. 3 and reported in Supplementary Table 4.

### Mediation

**Figure 4:**
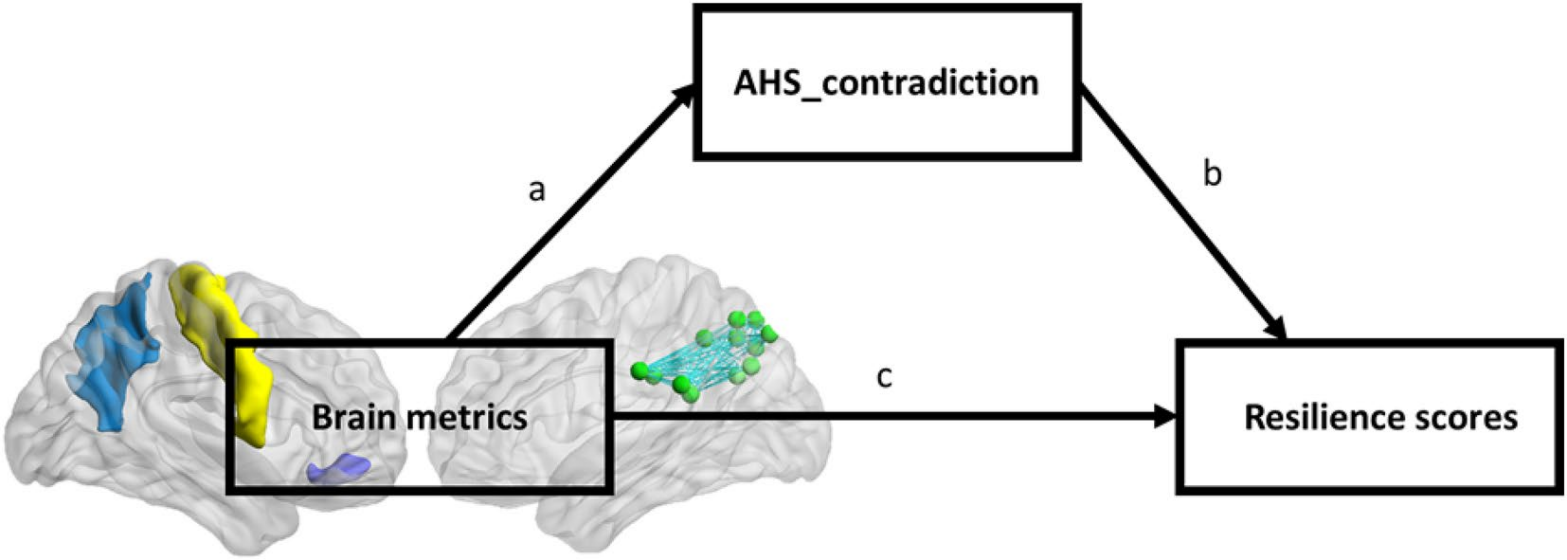
Mediation model of attitudes towards contradictions in relations between brain matrices and psychological resilience. AHS: Analysis-Holism Scale.

To further examine our hypothesis on the role of conflicting resolution in determining psychological resilience, we tested a parallel mediation model on attitudes towards contradictions across groups mediating the relationship between structural and functional brain matrices and resilience scores. The mediation model is visualized in Fig. 4. Mediation analysis revealed a significant indirect effect of attitudes toward contradictions on the links between brain metrics and psychological resilience. Specifically, the 95% bias-corrected confidence interval based on 5000 bootstrap samples revealed a significant specific indirect effect of LH_parORB, R_precentral, and R_precuneus in structural brain metrics on the resilience measure through AHS_contradiction as a mediator. Similarly, in functional brain metrics, controlC_PC showed a significant specific indirect effect on the resilience measure through AHS_contradiction as a mediator. All results were reported in Supplementary Table 5.

### General discussion

We investigated whether attitudes towards contradictions, as gauged by the AHS, facilitate resilience amid life's challenges by influencing the management and resolution of conflicts, a relationship that may be reflected in brain network patterns. The subscales used within the AHS to assess attitudes toward contradictions revealed discernible differences between the two thinking styles in question—formal logic and naïve dialecticism. Our findings indicated that scores related to naïve dialecticism are not only significantly higher but also exhibit a smaller standard deviation compared to those of formal logic. This pattern suggested a greater consistency among Asian participants who align with naïve dialecticism, implying that this cognitive style may be measured with greater reliability within our sample. Furthermore, our observations indicate a distinctive pattern: a significant and unique correlation exists between the dialectical thinking style, as measured by the AHS, and the Zhongyong thinking style, as denoted by the ZTS. This correlation aligns with the central hypothesis that the cognitive style characterized by an acceptance of complexity and a synthesis of varied perspectives—hallmarks of naïve dialecticism—correlates strongly with Zhongyong's holistic approach. These observations suggested that the differences in the attitude toward contradiction in levels of psychological resilience, indicating the possible role of attitudes towards contradictions (i.e., formal logic and naïve dialecticism) on the appraisal of the situation, may be related to resilience measures in the face of life's challenges and difficulties. To support this notion, we further examined the attitude toward contradiction differences in structural and functional MRI properties associated with brain cortices involved in conflict resolution. The group differences in structural and functional brain cortices, such as the inferior frontal area, parietal and anterior cingulate cortex, were commonly thought of as parts of brain networks for conflicting resolution. Structurally asymmetric between-group differences of brain volumetrics in these brain regions indicate right-hemispheric lateralization in different attitudes towards contradictions within cortical networks connectivity in dorsal attention network positively associated with psychological resilience estimates at the individual level, suggesting possible links of top-down conflict resolution process in psychological resilience. Mediation results highlighted that individuals with stronger attitudes toward resolving contradictions might have more psychological resilience. These attitudes toward contradictions may help individuals regulate the impact of conflicting information on their emotions and cognition, leading to better mental health and quality of life. The results also highlight the importance of frontoparietal brain networks for executive control in resolving conflicting information and regulating the impact of contradictions on psychological resilience. Our findings suggest a potential role of cortical conflicting resolution networks in attitudes towards contradictions and psychological resilience.

One's general perceptions and misperceptions of a challenging situation would lead to entirely different coping strategies for stressor events. Indeed, our findings observed that the differences in the attitude toward contradiction modulated levels of psychological resilience, indicating the possible role of thinking styles in the appraisal of the situation may be related to resilience estimates in facing life's challenges and difficulties. These observations suggested the potential role of attitudes towards contradictions as coping strategies in facing life difficulties or conflict situations. A previous patient study^43^ with lower extremity amputation reported that positive meaning aspects, such as improved attitudes towards life and independence, were noted in 49% of the 104 patients. They also found that positive meaning about amputation was linked to higher ratings of physical capabilities, better adjustment to physical limitations, and lower activity restriction. As such, thinking style does appear to be a key determinant of long-term adjustment to amputation and related face-of-life adversities. The ability to switch ways of thinking about how they perceive and interpret adverse events in their immediate and future lives^43^. For example, a child might see an obstacle as an insurmountable barrier or a transient challenge that offers a unique opportunity for learning, growth, and aptitude. When children have higher self-efficacy and a more vital internal locus of control, they are more likely to exhibit adaptive behaviors and attitudes that can lead to resilience in various areas of development^44,45^. These findings support our view^44^ of perception or cognitive appraisal of the situations about their later adaption into everyday life. According to a previous review, coping strategies often distinguish between confronting conflicts head-on and those that aim to reduce tension by avoiding confrontation with the conflict. Likewise, a recent large-scale study^46^ investigated brain structural correlates of parent–child relationships in eight thousand children. Their findings reported that high family conflict and low parental monitoring scores are associated with children’s behavioral problems and smaller cortical areas of the inferior frontal cortex, anterior cingulate cortex, and middle temporal gyrus. In our study, we observed the thinking style differences in structural and functional MRI properties associated with brain cortices involved in conflict resolution^47–49^, supporting our notion of the role of conflict resolution in the face of life difficulties or adversities.

In anticipation, our hypothesis posited that Zhongyong's holistic tendencies would differ in their correlation with naïve dialecticism versus formal logic. The data from our East Asian sample indeed reveal this to be the case, underscoring the cultural specificity of cognitive processes. The clarity of these correlations not only supports our initial hypothesis but also enriches the broader dialogue on how cognitive diversity, shaped by deep-seated cultural and philosophical beliefs, manifests in the ability to withstand and adapt to life's adversities. The ZTS^4^, derived from the core Confucian principle of pursuing balance and harmony, inherently involves a holistic consideration of multiple perspectives. Such a holistic tendency shares common ground with naïve dialecticism, a cognitive style characterized by a tolerance for contradiction and a propensity for seeking synthesis among opposing views. This investigation into the cognitive styles of East Asian populations reinforces the notion that a dialectical perspective, which emphasizes finding a middle way and favors balancing conflicting viewpoints^50^, may be inherently linked to resilience. In this context, incorporating the ZTS was to establish a foundational reference for our East Asian sample set. Thus, our research elucidates the multifaceted nature of cognitive approaches in navigating life’s adversities and their implications for psychological resilience.

Despite the fruitful findings above, this study found noteworthy variations in the social support scale of the QOL^22^ between attitudes toward contradictions, with Naïve Dialecticism exhibiting a correlation with higher social support levels. Consistent with this observation, the connection between the attitudes towards contradictory subscales of cognitive thinking style measurements and levels of psychological resilience was already established in this study. Prior studies have indicated that social support is a powerful predictor of psychological resilience^51,52^. The current study speculates that these different attitudes towards contradictions can lead to differences in social connections, which can ultimately impact psychological resilience. Together with prior studies^53^, we deem that Naïve Dialecticism was related to stronger social connections, which could clarify why it is linked to greater psychological resilience. Naïve Dialecticism accentuates the importance of contextual factors, which could help individuals to build stronger social connections and support networks. On the other hand, the Formal Logical thinking style may be less effective in forming these connections, as it prioritizes rules and principles. These findings suggest that distinct attitudes towards contradictions could influence an individual's psychological resilience by affecting their social connections. It underscores the significance of considering cultural and contextual factors when examining these complex concepts. It also implies that Naïve Dialecticism could be crucial in building strong social connections and promoting psychological resilience.

In this study, Control-A, -B, and -C resemble subnetworks of the frontoparietal brain regions. These brain regions and networks are key for executive control of cognition and emotion in conflicting task-irrelevant information. The frontoparietal spans frontal, parietal, and cingulate brain regions associated with executive functions^54,55^. These regions formed as brain networks subserve conflicting resolution and encompass across brain regions, including the inferior frontal cortex (IFC), superior parietal cortex (SPC), superior occipital cortex (SOC), and right anterior cingulate cortex (ACC)^47–49^. Structurally asymmetric between-group differences with different attitudes towards contradictions of brain volumetrics in these brain regions indicate right-hemispheric lateralization in right-handed healthy young adults. According to Roger's hypothesis^56^, in right-handers, the right hemisphere is responsible for maintaining stability and reacting quickly to unexpected stimuli from both the external and internal environment. On the other hand, the left hemisphere is responsible for predicting future events and maintaining precise and efficient movements in predictable situations. Specifically, from a motor control perspective, the right hemisphere is specialized in using impedance control mechanisms to maintain stable positions and velocities in the face of unpredictable events and to ensure accuracy and stability in steady-state postures^57^. However, a modified version of the hemispheric lateralization hypothesis^58^ suggests that the right hemisphere is primarily responsible for emotion-related processes, while the left hemisphere plays a lesser role. This revised view argues that the right hemisphere is naturally biased toward emotional perception^59^. Interestingly, according to the approach-withdrawal hypothesis^60^, the right hemisphere, particularly the frontal lobe, is activated in response to emotions that elicit avoidance behavior. The left hemisphere is activated in response to emotions that encourage approach behavior. In sum, this hypothesis on right-hemispheric lateralization in right-handers could be speculated as emotional-related behavior, especially in avoidance behavior. Our findings indicate attitudes towards contradictions differences in brain structural and functional brain cortices in relation to conflict resolution networks may contribute to avoidance behavior in conflicting or difficult situations in life.

Regarding functional brain dynamics, we observed cortical network connectivity in the dorsal attention network positively associated with psychological resilience measures at the individual level, suggesting possible links to the top-down conflict resolution process in psychological resilience. The dorsal attention network comprises frontal and parietal regions involved in goal-directed processing related to visual attention^61^. Specifically, the dorsal attention network includes the frontal eye fields, superior parietal lobule, and intraparietal sulcus^33,61,62^. The critical brain regions within the dorsal attention network are also spanned over the brain regions related to processing conflict resolution^47–49^ such as IFC, SPC, SOC, and right ACC. A previous study^63^ examined the influence of emotion on executive control by examining the flanker conflict effect (incongruent-congruent) with emotional and neutral word stimuli. The findings revealed that the ventral ACC responds to cognitive conflict, as indicated by using stimuli with different colors, but only in the presence of emotional stimuli with negative valence. The combination of reduced reaction time in the conflict condition and increased activation in the ventral ACC suggests that emotion may substantially impact conflict resolution more than conflict monitoring. In line with our previous discussion on right hemisphere lateralization in brain structural and functional cortices, a recent study^64^ has shown the right-hemisphere superiority of this dorsal attention network. Our study found that thinking style differences in the shared brain structural and functional brain cortices are associated with levels of psychological resilience, especially in conflict resolution for the cognitive control of emotions.

There are some uncertainties and limitations that should be considered. Firstly, this study’s sample consisted of only healthy young adults, which may limit the generalizability of the findings to other populations, age groups, and settings. We recognize that our study might be limited by the sample size and the estimation of effect size. The absence of large-scale datasets in this specific research area poses a challenge to achieving the ideal statistical power. Future studies could address this limitation by recruiting a more diverse sample to improve the generalizability of the findings. Secondly, the cross-sectional design of this study does not allow for causal inferences, and longitudinal studies are needed to confirm the relationship between attitudes toward contradictions and psychological resilience. Thirdly, using self-reported measures for cognitive thinking styles and psychological resilience may be subject to bias and may not accurately reflect actual cognitive processes or resilience in real-life situations. Specifically, we acknowledge the limitations in operationalizing psychological resilience primarily through self-report questionnaires. Kalisch et al. (2017) suggested that resilience is best measured by tracking individuals' functioning levels after exposure to life adversities^65^, which our current study design does not encompass. Kalisch and colleagues argue that resilience is best understood by examining variations in markers and competencies post-adversity, emphasizing the importance of situational processes in resilient performance^65,66^. This approach aligns with the conceptualization of resilience as not only the maintenance but also the rapid recovery of mental health and psychosocial functioning during and after times of adversity^65–67^. Acknowledging these perspectives, our study could benefit from integrating measures that capture these dynamic changes, thereby providing a more comprehensive understanding of psychological resilience. Accordingly, future research could benefit from employing longitudinal designs that track individuals' mental health trajectories over time, post-adversity. Lastly, this study's reliance on self-report measures and the use of a median split to categorize participants may affect the reproducibility of the results, primarily since this study's limitations surrounding the dichotomy of East–West cultural thinking styles in East Asian societies must be acknowledged. Nonetheless, examining these differences within the context of East Asia alone diminishes the potential for cultural biases and assumptions, providing a more nuanced and culturally specific understanding of the biases and attitudes towards the conflict in these societies.

## Conclusion

Together, our findings contributed to cumulative theoretical knowledge in social psychology, social cognition, and attitudes by exploring the relationship between attitudes toward contradictions, conflict resolution-related cortical brain networks, and psychological resilience at the neural level using structural and functional MRI in different attitudes toward contradiction groups. We built on and extended commonly accepted theoretical frameworks in attitudes and social cognition by examining the role of cortical conflict resolution networks between attitudes towards contradictions and psychological resilience. It also challenged these frameworks by suggesting potential links between conflict resolution and psychological resilience in the frontoparietal network. These findings support the hypothesis that cognitive thinking style measurements could be linked to levels of psychological resilience in attitudes toward contradictions. The differences in the attitude toward contradiction in levels of psychological resilience suggested the possible role of thinking styles in the appraisal of the situation, which is also in line with our conceptual model of psychological resilience^68^.

We highlighted executive control's importance in regulating contradictions' influence on psychological resilience, emphasizing the need to recognize realistic obstacles to a successful resolution. We also suggested the potential role of thinking styles as coping strategies in facing life difficulties or conflict situations and highlighted the importance of contextual factors in building stronger social connections and support networks. In sum, we provide valuable insights into the neural mechanisms underlying the relationship between attitude towards contradictions, conflict resolution-related cortical brain networks, and psychological resilience. Regarding the implications, our findings can be helpful in developing interventions to help individuals deal with adversity and develop resilience by focusing on cognitive thinking styles and conflict resolution strategies.

### Supplementary Information

Supplementary Information.

## Data Availability

The datasets used and analyzed during the current study available from the corresponding author on reasonable request.
