# Supplementary material for: The role of attitudes towards contradiction in psychological resilience: the cortical mechanism of conflicting resolution networks
Source: Sci Rep. 2024 Jan 18;14:1669. doi: 10.1038/s41598-024-51722-3 (PMC10796669; doi:10.1038/s41598-024-51722-3)
Supplement: Supplementary file 1 — Supplementary Information. [file 41598_2024_51722_MOESM1_ESM.docx]

**Supplementary file**

Table 1. Group Differences between structural brain metrics in the left and right hemispheres.

|  | Left hemisphere | | Right hemisphere | |
| --- | --- | --- | --- | --- |
|  | *t*-value | *p*-value | *t*-value | *p*-value |
| Banks of the Superior Temporal Sulcus | 0.746 | 0.457 | 1.441 | 0.151 |
| Caudal Anterior Cingulate | 0.757 | 0.450 | 0.806 | 0.421 |
| Caudal Middle Frontal | 1.615 | 0.108 | 0.932 | 0.353 |
| Cuneus | 0.385 | 0.701 | 0.483 | 0.630 |
| Entorhinal | 1.807 | 0.072 | 0.746 | 0.457 |
| Fusiform | 0.551 | 0.582 | 0.087 | 0.931 |
| Inferior Parietal | 0.896 | 0.371 | 2.162 | 0.032 |
| Inferior Temporal | 0.878 | 0.381 | 1.975 | 0.050 |
| Isthmus Cingulate | 0.857 | 0.392 | 1.527 | 0.129 |
| Lateral Occipital | 1.789 | 0.075 | 0.799 | 0.426 |
| Lateral Orbitofrontal | 1.403 | 0.163 | 1.175 | 0.242 |
| Lingual | 0.844 | 0.400 | 1.383 | 0.168 |
| Medial Orbitofrontal | 0.384 | 0.702 | 0.219 | 0.827 |
| Middle Temporal | 1.367 | 0.173 | 2.065 | 0.040 |
| Parahippocampal | 1.667 | 0.097 | 1.270 | 0.206 |
| Paracentral | 1.872 | 0.063 | -1.046 | 0.297 |
| Pars Opercularis | 1.885 | 0.061 | 2.104 | 0.037 |
| Pars Orbitalis | 3.920 | 0.000 | 2.317 | 0.022 |
| ParsTriangularis | -0.007 | 0.995 | 0.937 | 0.350 |
| Pericalcarine | -0.798 | 0.426 | 0.199 | 0.843 |
| Postcentral | 0.867 | 0.387 | 1.659 | 0.099 |
| Posterior Cingulate | 1.462 | 0.145 | -0.476 | 0.635 |
| Precentral | 1.796 | 0.074 | 2.725 | 0.007 |
| Precuneus | 1.069 | 0.287 | 2.461 | 0.015 |
| Rostral Anterior Cingulate | 1.141 | 0.255 | 1.304 | 0.194 |
| Rostral Middle Frontal | 1.231 | 0.220 | 1.452 | 0.148 |
| Superior Frontal | 1.051 | 0.295 | 0.622 | 0.535 |
| Superior Parietal | 0.073 | 0.942 | 1.356 | 0.177 |
| Superior Temporal | 0.893 | 0.373 | 1.585 | 0.115 |
| Supramarginal | 1.868 | 0.063 | 0.972 | 0.333 |
| Frontal Pole | 1.066 | 0.288 | 1.322 | 0.188 |
| Temporal Pole | 1.267 | 0.207 | 0.832 | 0.406 |
| Transverse Temporal | 0.142 | 0.887 | 0.040 | 0.968 |
| Insula | 1.348 | 0.179 | 1.110 | 0.269 |

Table 2. Group Differences between functional brain metrics in the left and right hemispheres.

|  | PC | | WMD | |
| --- | --- | --- | --- | --- |
|  | TracePilliai | *p*-value | TracePilliai | *p*-value |
| Central Visual | 0.121 | 0.673 | 0.217 | 0.029 |
| Peripheral Visual | 0.120 | 0.624 | 0.130 | 0.569 |
| SomatoMotor A | 0.184 | 0.825 | 0.253 | 0.268 |
| SomatoMotor B | 0.170 | 0.572 | 0.142 | 0.823 |
| Dorsal Attention A | 0.143 | 0.613 | 0.207 | 0.108 |
| Dorsal Attention B | 0.224 | 0.029 | 0.078 | 0.979 |
| Salience/Ventral Attention A | 0.228 | 0.233 | 0.333 | 0.002 |
| Salience/Ventral Attention B | 0.095 | 0.510 | 0.073 | 0.784 |
| Limbic B | 0.058 | 0.441 | 0.035 | 0.880 |
| Limbic A | 0.062 | 0.649 | 0.072 | 0.498 |
| Control A | 0.114 | 0.739 | 0.150 | 0.362 |
| Control B | 0.152 | 0.399 | 0.142 | 0.507 |
| Control C | 0.065 | 0.515 | 0.129 | 0.030 |
| Default A | 0.141 | 0.915 | 0.204 | 0.419 |
| Default B | 0.184 | 0.492 | 0.193 | 0.412 |
| Default C | 0.049 | 0.826 | 0.055 | 0.750 |
| Temporal Parietal | 0.103 | 0.337 | 0.065 | 0.809 |

Table 3. Correlation (Pearson’s *r* value) between resilience measures and demographic variables by group

|  | RSA_ps | | RSA_fc | | RSA_sr | | RSA_sc | | RSA_fs | | RSA_total | |
| --- | --- | --- | --- | --- | --- | --- | --- | --- | --- | --- | --- | --- |
|  | Logic | Naïve | Logic | Naïve | Logic | Naïve | Logic | Naïve | Logic | Naïve | Logic | Naïve |
| MoCA | 0.009 | 0.018 | -0.119 | 0.106 | 0.138 | 0.134 | 0.063 | 0.082 | -0.100 | 0.142 | 0.003 | 0.131 |
| BDI | -0.594*** | -0.645*** | -0.219* | -0.168 | -0.380*** | -0.122 | -0.140 | -0.192 | -0.553*** | -0.435*** | -0.547*** | -0.419*** |
| QOL_Phy | 0.441*** | 0.548*** | 0.141 | 0.317** | 0.329** | 0.269* | 0.253* | 0.332** | 0.413*** | 0.449*** | 0.451*** | 0.515*** |
| QOL_Psy | 0.482*** | 0.720*** | 0.068 | 0.369** | 0.378*** | 0.378*** | 0.389*** | 0.343*** | 0.434*** | 0.630*** | 0.493*** | 0.660*** |
| QOL_Soc | 0.476*** | 0.453*** | 0.129 | 0.288** | 0.634*** | 0.510*** | 0.467*** | 0.239* | 0.342*** | 0.396*** | 0.602*** | 0.523*** |
| QOL_Env | 0.446*** | 0.402*** | 0.090 | 0.453*** | 0.359*** | 0.407*** | 0.171 | 0.210* | 0.329** | 0.413*** | 0.407*** | 0.519*** |
| ZTS | 0.209 | 0.420*** | -0.012 | 0.265* | 0.386*** | 0.261* | 0.343** | 0.191 | 0.221* | 0.344*** | 0.330** | 0.404*** |

Note: “*”: *p* < 0.05; “**”: *p* < 0.01; “***”: *p* < 0.001; RSA=Resilience Scale for Adults (ps=personal strength; fc=family cohesion; sr=social resource; sc=social competence; fss=future structured style); MoCA=Montreal Cognitive Assessment; BDI-II=Beck’s Depression Inventory-II; QOL=Quality of life (Phy=physical health; Men=mental health; Soc=social relations scale; Env=environmental health scale; ZTS =Zhongyong Thinking Scale).

Table 4. Correlations between resilience measures and brain metrices by group

|  | RSA_ps | | RSA_fc | | RSA_sr | | RSA_sc | | | RSA_fs | | | RSA_total | |
| --- | --- | --- | --- | --- | --- | --- | --- | --- | --- | --- | --- | --- | --- | --- |
|  | Logic | Naïve | Logic | Naïve | Logic | Naïve | Logic | Naïve | Logic | | Naïve | Logic | | Naïve |
| parORB_L | 0.011 | 0.022 | -0.004 | 0.155 | 0.025 | 0.036 | -0.106 | 0.058 | -0.084 | | 0.073 | -0.034 | | 0.092 |
| infP_R | 0.024 | -0.067 | 0.071 | 0.098 | 0.076 | 0.138 | 0.110 | 0.057 | 0.017 | | -0.086 | 0.088 | | 0.044 |
| midT_R | 0.108 | -0.136 | 0.136 | 0.113 | 0.095 | 0.121 | -0.070 | 0.027 | 0.030 | | -0.036 | 0.103 | | 0.029 |
| parOPC_R | 0.171 | -0.075 | 0.099 | 0.209* | 0.121 | -0.093 | 0.143 | -0.223* | 0.096 | | -0.087 | 0.183 | | -0.066 |
| parORB_R | -0.089 | 0.071 | 0.119 | -0.005 | -0.041 | 0.028 | 0.250* | 0.058 | -0.124 | | 0.128 | -0.088 | | 0.072 |
| precentral_R | -0.025 | 0.062 | -0.026 | 0.166 | 0.073 | -0.016 | 0.127 | 0.05 | 0.108 | | 0.056 | 0.067 | | 0.083 |
| precuneus_R | 0.125 | -0.080 | 0.165 | 0.118 | 0.132 | -0.036 | 0.088 | -0.053 | 0.026 | | -0.069 | 0.167 | | -0.031 |
| DorAttB_WMD | 0.206 | -0.008 | 0.363*** | 0.065 | 0.152 | 0.152 | 0.186 | 0.119 | 0.220* | | 0.029 | 0.333** | | 0.099 |
| CenVisu_PC | -0.063 | -0.068 | -0.057 | 0.088 | -0.180 | -0.115 | -0.138 | -0.084 | -0.095 | | -0.058 | -0.158 | | -0.066 |
| SaVenAtt_PC | 0.011 | -0.029 | -0.004 | 0.034 | -0.094 | -0.079 | 0.038 | -0.108 | 0.072 | | -0.079 | -0.007 | | -0.068 |
| ControlC_PC | 0.064 | 0.118 | -0.080 | 0.026 | -0.054 | -0.016 | -0.121 | 0.001 | -0.166 | | 0.050 | -0.097 | | 0.048 |

Note: “*”: p < 0.05; “**”: p < 0.01; “***”: *p* < 0.001

Table 5. Mediation coefficient values for different models, respectively.

| Brain metrics | a-path coefficient | mediator | b-path coefficient | Outcome | a x b path | Lower-bond | Upper-bond |
| --- | --- | --- | --- | --- | --- | --- | --- |
| LH_parORB | -0.2886 | AHS_contradiction | 0.3830 | RSA_FC | -0.1105 | -0.0442 | -0.1913 |
| LH_parORB | -0.2886 | AHS_contradiction | 0.1813 | RSA_total | -0.0523 | -0.0046 | -0.1227 |
| R_precentral | -0.1850 | AHS_contradiction | 0.3751 | RSA_FC | -0.0694 | -0.0126 | -0.1297 |
| R_precuneus | -0.2033 | AHS_contradiction | 0.3933 | RSA_FC | -0.0799 | -0.0139 | -0.1607 |
| controlC_PC | -0.1482 | AHS_contradiction | 0.3649 | RSA_FC | -0.0541 | -0.0012 | -0.1217 |

**Figure 1.** Box plot for two scores distribution of the formal logic and naïve dialecticism


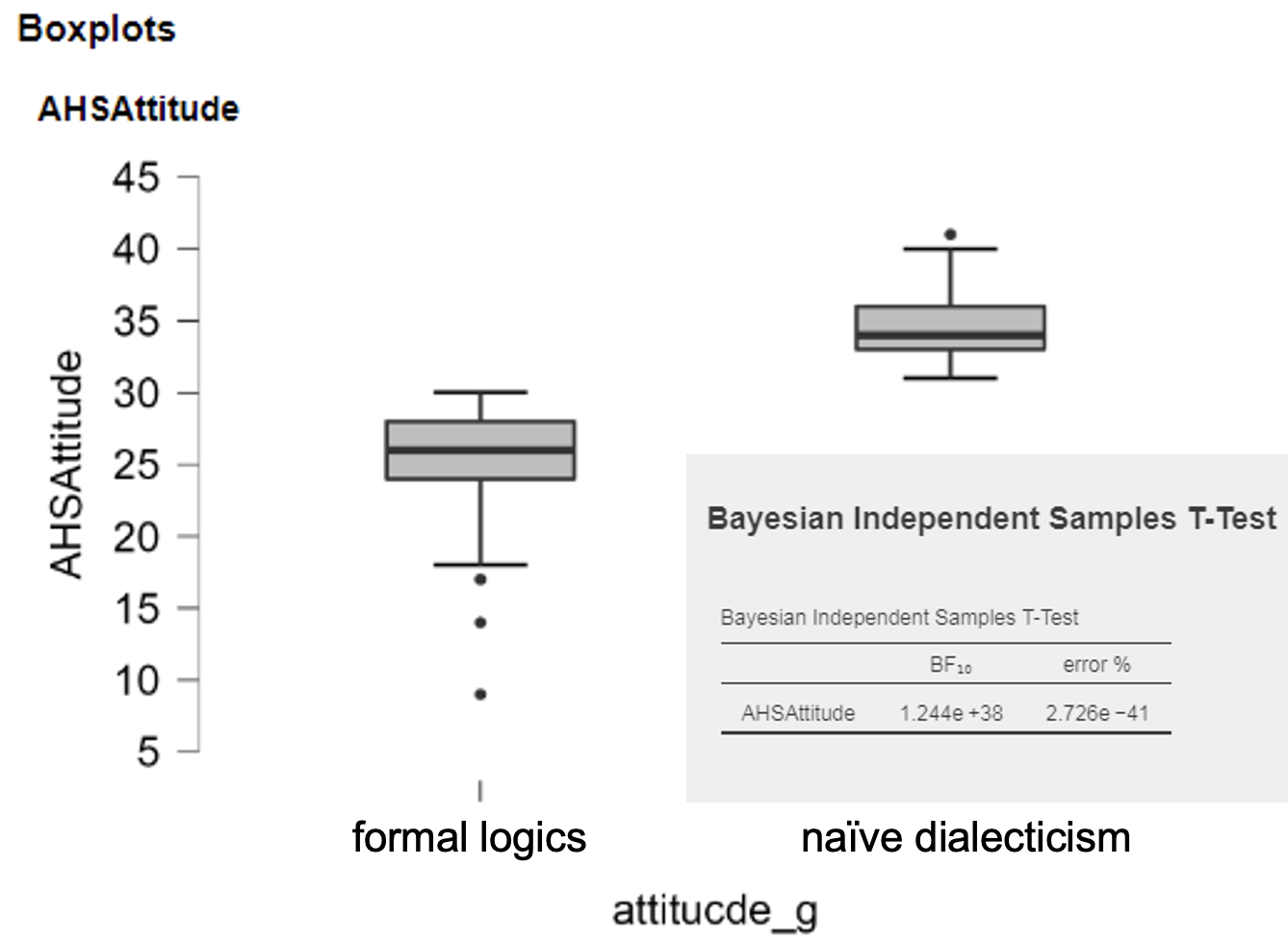


**Figure 2.** Descriptive statistics with distribution plots on the formal logic and naïve dialecticism

**
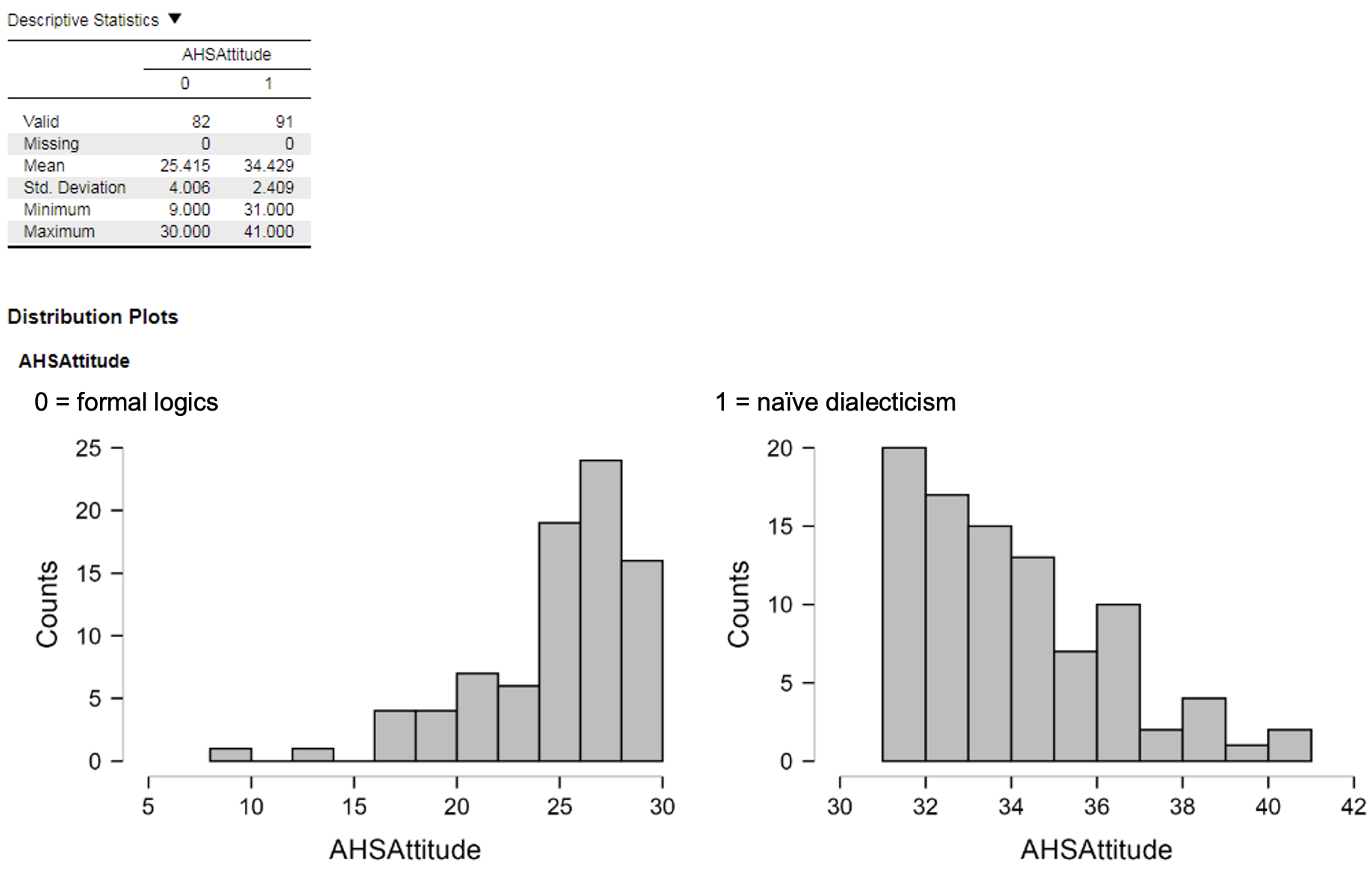
**
